# Metabolic Health, Overweight or Obesity, and Depressive Symptoms among Older Australian Adults

**DOI:** 10.3390/nu16070928

**Published:** 2024-03-23

**Authors:** Jacob Opio, Katie Wynne, John Attia, Christopher Oldmeadow, Stephen Hancock, Brian Kelly, Kerry Inder, Mark McEvoy

**Affiliations:** 1 School of Medicine and Public Health, University of Newcastle, University Drive, Callaghan, NSW 2308, Australia; jacob.opio@uon.edu.au (J.O.); katiejane.wynne@health.nsw.gov.au (K.W.); john.attia@newcastle.edu.au (J.A.); christopher.oldmeadow@hmri.org.au (C.O.); brian.kelly@newcastle.edu.au (B.K.); 2 Diabetes and Endocrinology, John Hunter Hospital, Lot 1 Kookaburra Circuit, New Lambton Heights, NSW 2305, Australia; 3 Hunter Medical Research Institute, School of Medicine and Public Health, University of Newcastle, University Drive, Callaghan, NSW 2308, Australia; stephen.hancock@newcastle.edu.au (S.H.); kerry.inder@newcastle.edu.au (K.I.); 4 School of Nursing and Midwifery, College of Health Medicine and Wellbeing, University of Newcastle, University Drive, Callaghan, NSW 2308, Australia; 5 La Trobe Rural Health School, College of Science, Health and Engineering, La Trobe University, Edwards Road, Flora Hill, VIC 3552, Australia

**Keywords:** obesity, overweight, normal weight, metabolic health, depression, depressive symptoms

## Abstract

Background: The relationship between overweight or obesity and depressive symptoms in individuals with or without cardio-metabolic abnormalities is unclear. In a cross-sectional study we examined the odds of experiencing depressive symptoms in overweight or obese older adults with or without metabolic abnormalities. Methods: The participants included 3318 older adults from the Hunter Community Study Cohort with a Body Mass Index (BMI) ≥ 18.5 kgm^2^, stratified by BMI and metabolic health risk. Obesity was defined as BMI ≥ 30 kgm^2^ and metabolically healthy as the absence of metabolic risk factors, according to International Diabetic Federation criteria for metabolic syndromes. Moderate to severe depressive symptoms were defined as a Centre for Epidemiological Studies Depression Scale (CES-D) score ≥ 16. Results: Compared to the metabolically healthy normal weight (MHNW) group, the odds of experiencing moderate/severe depressive symptoms were higher in those classified as a metabolically unhealthy normal weight (MUNW) (odds ratio (OR) = 1.25, 95% Confidence Interval (CI): 0.76–2.06) or metabolically unhealthy obesity (MUO) (OR = 1.48, 95% CI: 1.00–2.19), but not in those classified as metabolically unhealthy overweight (MUOW) (OR = 0.96, 95% CI: 0.63–1.45), metabolically healthy overweight (MHOW) (OR = 0.80, 95% CI: 0.51–1.26), and metabolically healthy obesity (MHO) (OR = 1.03, 95% CI: 0.65–1.64). Compared with MHNW males, the odds of moderate/severe depressive symptoms were increased in all other BMI category–metabolic health groups for males and females. Limitations: Our relatively small sample size and cross-sectional design did not allow us to robustly establish causality. Conclusion: The odds of experiencing moderate/severe depressive symptoms were increased in metabolically unhealthy older adults regardless of normal weight or obesity, with the odds of having moderate/severe depressive symptoms being higher in females than in males.

## 1. Introduction

The global prevalence of obesity has reached an epidemic proportion, with a WHO report showing about 2 billion adults classified as being overweight and 650 million crossing the obesity threshold [1]. The previous global prevalence of obesity was equally high [2]. Obesity is associated with an increased risk of metabolic syndrome, type 2 diabetes, and cardiovascular disease [3,4]. The current estimates of the worldwide prevalence of depression indicate that approximately 280 million people or more suffer from a depressive disorder. The global burden of disease (GBD) study conducted by the WHO showed an increasing trend in the burden of disability due to depressive disorders. Depressive disorders were ranked 13th among the top leading causes of disability-adjusted life years (DALYs) and 2nd in the years of healthy life lost due to disability (YLDs) in 2019 [5]. The risk of experiencing depressive symptoms involves a complex interaction of social, psychological, and biological factors including chronic diseases, patterns of diet and alcohol use, and genetic factors [6]. Overweight and obesity are being investigated for their potential for increasing the risk of developing clinically significant depressive symptoms as well as other established sequalae such as metabolic syndrome. Overweight or obesity is defined as the abnormal or excessive accumulation of body fat that leads to health risks, assessed using any one of the following measures: (1) BMI (obesity ≥ 30 kgm^2^, overweight—25–29.9 kgm^2^), (2) Percentage body fat (female: obesity ≥ 35%, overweight—30–35%; male: obesity ≥ 30%, overweight—25–29%), or (3) Abdominal obesity (female waist circumference ≥ 88 cm, male waist circumference ≥ 102 cm) [7,8].

Overweight and obesity lead to pathogenic adiposity and adipose tissue endocrine and immune dysfunction, which contribute to metabolic diseases, such as type 2 diabetes mellitus, hypertension, dyslipidaemia, cardiovascular disease, and cancer. Metabolic syndrome is not a disease but a descriptive clustering of atherosclerotic risk factors [7]. The diagnostic criteria for metabolic syndrome include three or more of the following five cardiovascular disease risk factors:

1. Abdominal obesity (waist circumference ≥ 102 cm in males, ≥88 cm in females),
2. Triglycerides ≥ 1.7 mmol/L,
3. High-Density Lipoprotein cholesterol (HDL) < 1.04 mmol/L in males, <1.30 mmol/L in females.
4. Blood pressure ≥ 130/85 mmHg,
5. Fasting glucose ≥ 5.6 mmol/L.

Several studies have demonstrated a positive association between overweight, obesity, and depressive symptoms or disorders. One systematic review and five different meta-analyses performed between 2008 and 2015 all showed a positive association between obesity and depressive symptoms, with an odds ratio (OR) range of 1.18 to 1.73 [9,10,11,12,13,14]. These studies used a variety of scales, instruments, and diagnoses with varying terminologies to define depression or depressive symptoms, including a Centre for Epidemiological Studies Depression Scale (CES-D) score of 16 or greater, which was used to define moderate to severe depressive symptoms [15]. Two of the studies found a bidirectional association between obesity and depression, assessed using the Diagnostic and Statistical Manual of Mental Disorders, 4th Edition (DSM-IV) criteria for a major depressive disorder (MDD) or depressive symptoms scales including a CES-D score of 16 or more [11,14]. Obesity at baseline increased the odds of experiencing moderate to severe depressive symptoms or an MDD (OR = 1.55; 95% CI: 1.22–1.98; *p* < 0.001), and moderate to severe depressive symptoms or a DSM-IV diagnosis of MDD increased the odds of a person developing obesity (OR = 1.58; 95% CI, 1.33–1.87; *p* < 0.001), as reported by Luppino et al. [11]. In another recent study, the risk of obesity was increased in adults with moderate to severe depressive symptoms or a DSM-IV diagnosis of MDD (RR = 1.37; 95% CI, 1.17–1.48), and the risk of moderate to severe depressive symptoms or an MDD was increased in adults with obesity (RR = 1.18; 95% CI, 1.0–1.35). The association was stronger in the direction of moderate to severe depressive symptoms/MDD to obesity and more noticeable in young and middle-aged women, as recently reported by Mannan et al. [14]. The association between obesity and moderate to severe depressive symptoms/MDD was more pronounced in women in three studies with ORs ranging from 1.26 to 1.37 [10,13,14].

Several studies have also reported a positive association between metabolic syndrome and depression. A systematic review and meta-analysis reported a bidirectional association between depression assessed using the DSM-IV criteria for the diagnosis of MDD, or self-report symptom scales including a CES-D score of 16 or more, and metabolic syndrome, defined according to NECP ATP-III or IDF criteria, with increased odds of an existing MDD or high depressive symptoms in participants with metabolic syndrome (OR = 1.49; 95% CI, 1.19–1.89) and increased odds of developing metabolic syndrome in persons with an existing MDD or high depressive symptoms (OR = 1.52; 95% CI, 1.20–1.91) in a study by Pan et al. [16]. Two other studies, one longitudinal study [17] and another cross-sectional study [18], demonstrated a positive association between metabolic syndrome, defined by NCEP ATP-III or IDF criteria, and comorbid anxiety and depressive symptoms, assessed using the Hospital Anxiety and Depression Scale (OR = 3.84; 95% CI, 1.26–11.71), but not when anxiety or moderate to severe depressive symptoms were assessed independently using the CES-D scale.

While obesity and metabolic syndrome are clearly associated with moderate to severe depressive symptoms assessed using the CES-D scale [17], there is a subgroup of obese individuals referred to as having metabolically healthy obesity (MHO), who have minimal or no metabolic abnormalities and constitute about 10 to 40% of the population with obesity, depending on the definition of metabolic health used [19]. Few studies have investigated the relationship between MHO and depressive symptoms. Two cross-sectional studies carried out in 2012 and 2015 and a longitudinal study in 2017 all found no association between MHO and moderate to severe symptoms of depression, assessed using the CES-D scale [20,21,22]. These cross-sectional studies defined metabolic health as the presence of one or fewer metabolic risk factors, whereas metabolic health in the longitudinal study was defined as the absence of any metabolic risk factors. The above findings were consistent with findings from a systematic review which concluded that MHO was not associated with depressive symptoms when using the CES-D scale. Nevertheless, metabolically unhealthy obesity (MUO) (i.e., those with ≥1 metabolic risk factors and obesity) was associated with an increased risk of depressive symptoms, as recently reported by Malmir et al. [23]. However, a pooled analysis of eight cross-sectional studies found increased odds of moderate to severe depressive symptoms, assessed using standardised scales including the CES-D scale, in participants categorised as MHO, defined as having ≤1 risk factor [24]. All the studies above found increased odds of moderate to severe depressive symptoms in participants categorised as MUO.

There are no published studies from Australia, and very few internationally, that investigate the relationships between metabolic health, overweight, obesity, and depressive symptoms. There is also increased interest in the group of individuals categorised as having overweight or obesity with no cardiometabolic risk factors, and the evidence examining the association between MHO and clinically significant depressive symptoms is inconsistent.

Given that overweight/obesity and metabolic disturbances are common in older populations, this study therefore aimed to examine the cross-sectional association between overweight/obesity with and without cardiometabolic abnormality and moderate to severe depressive symptoms in a cohort of older Australian men and women.

## 2. Methods

### 2.1. Sample

The findings of this investigation are a secondary data analysis of the baseline data from the Hunter Community Study (HCS). The HCS is a population-based cohort study of community-dwelling men and women aged 55–85 years, living in Newcastle, New South Wales (NSW), Australia. A detailed description of the study methods has been published [25]. Briefly, participants who were contacted from December 2004 to December 2007 were randomly selected from the NSW State electoral roll. Being listed on the Australian electoral roll is compulsory, and its contents are estimated to be 93.6% complete. The study used a modified Dillman recruiting strategy [26], in which selected persons had two letters of introduction and an invitation posted to them. No response to the initial postal contact was followed by telephone contact from the HCS research team. Non-responders consisted of people who could not be contacted after five attempts. Non-English-speaking people and those residing in residential aged-care facilities were excluded. 

Participants were asked to complete self-administered questionnaires and attend a clinic to undertake a series of clinical assessments. They were also asked for their consent to link their questionnaire data to their Medicare Australia data (Medicare and Pharmaceutical Benefit Scheme (PBS)) so that future health events and health services interactions could be determined. There were 3318 participants who responded to the baseline questionnaires, a response rate of 44.5%. Responders and non-responders were similar in their gender profile, but the responders were slightly younger in age. The HCS participants’ profiles reflected that of the Hunter, NSW, and Australian population for gender and marital status; however, the cohort was slightly younger compared to the national and state populations.

This current analysis excluded participants who were underweight (BMI < 18.5 kgm^−2^), as the focus of this investigation was on overweight/obese adults compared to normal-weight adults, and the number of people in the underweight group was too small to provide a meaningful analysis. The study was conducted according to the guidelines laid down in the Declaration of Helsinki; all participants provided informed consent, and all procedures involving research study participants were approved by the University of Newcastle’s Human Research Ethics committee (code: H-820-0504).

### 2.2. Exposure Measurement

Metabolically healthy was defined as the absence of any of the following cardiometabolic risk factors, consistent with the International Diabetes Federation’s metabolic syndrome criteria [27]: triglyceride concentration ≥ 1.7 mmol/L; HDL cholesterol concentration < 1.0 mmol/L in men or <1.3 mmol/L in women, or lipid-lowering medication use; blood pressure ≥ 130/85 mmHg or antihypertensive medication use; and fasting glucose concentration ≥ 5.6 mmol/L or self-reported diabetes. Participants with no metabolic risk factors were classified as metabolically healthy. Their BMI was categorised according to the World Health Organisation (WHO) criteria [28]. Subjects were classified, according to their BMI, as normal weight (18.5–24.9 kg/m^2^), overweight (25–29.9 kg/m^2^), or obese (≥30.0 kg/m^2^), and these were combined with each person’s metabolic health status (i.e., metabolically healthy or metabolically unhealthy) to create the following six BMI category–metabolic health groups: MHNW, MHOW, MHO, MUNW, MUOW, and MUO.

### 2.3. Outcome Measurement

The primary outcome for this study is depressive symptoms, which were measured using the CES-D scale, with a score ≥16 defined as the presence of moderate to severe depressive symptoms. The CES-D measures positive and depressive affects, interpersonal relationships, and somatic complaints via a 20-item scale with a score spanning 0–60, where higher scores reflect greater depressive symptoms [15]. This scale is validated to be used in older-population-based cohorts [29], and there have been multiple studies that have used this threshold to indicate the likelihood of a depressive disorder [22,30].

### 2.4. Measurement of Potentially Confounding Variables

Based on previous research on the association between MHOW or MHO and depressive symptoms, directed acyclic graphs (DAGs) [31] were used to determine the confounding variables controlled for in this analysis, using contextual expert knowledge as well as the literature to inform the causal structure of the DAG. The variables considered as potential confounding variables included age, gender, diet quality, household income, education, smoking, alcohol intake, antidepressant medication use, and physical activity level. Based on the constructed DAG from DAGitty v 3.1 [32] [see Appendix A], the minimum adjustment set to control for confounding in this analysis was age, gender, education, smoking, alcohol intake, diet quality, and physical activity level. Age, gender, and education were based on the information provided in self-administered questionnaires. Education was classified according to the highest level of education attained: classified as having completed primary and secondary schooling or a trade qualification, university, or other tertiary-level study. Physical activity was measured by recorded step counts, using a pedometer worn by participants for seven consecutive days during waking hours, and was reported as their mean number of steps per day. Smoking frequency was measured by the number of cigarettes per day and reported as the respondent being a never-, ever-, or current smoker, and alcohol consumption was measured by the number of standard drinks per day, defined according to Australian National Health and Medical Research Council (NHMRC) guidelines, and reported as the mean number of a respondent’s standard alcohol drinks per day [33]. Dietary intake at baseline was measured by a self-administered 145-item semi-quantitative Food Frequency Questionnaire (FFQ) that was previously validated against four-day weighed food records in older Australian adults participating in the Blue Mountain Eye Study [34]. The participants were required to indicate their usual consumption frequency of food items, with a nine-category frequency scale ranging from ‘never’ to ‘four or more times per day’, during the previous 12 months [34]. Diet quality was measured using the Australian Recommended Food Score (ARFS) [35]. The ARFS was calculated from the FFQ. The ARFS focuses on the variety of foods consumed within each recommended food group of the Australian Dietary Guidelines [36]. It contains seven categories—vegetables, fruit, protein foods, grains, dairy products, fats, and alcohol [37]. One point is usually assigned to most of these foods if they are consumed at least once per week, depending on the Australian Dietary Guideline [36,38]. The ARFS is the sum of each item, and the maximum score is 74; the higher the ARFS score, the better the diet quality.

### 2.5. Study Design and Statistical Analysis

Participants were categorised into six groups according to their BMI and metabolic health status, as described above. A chi-squared test and an ANOVA with a Bonferroni test were used to examine the differences in the baseline characteristics of the different groups, with a *p*-value less than 0.05 considered statistically significant. Multiple logistic regression was used to estimate the OR and 95% CI for the association between each of the six BMI category–metabolic health groups and depressive symptoms, using the cross-sectional data at baseline. The MHNW group was used as the reference group. For each BMI category–metabolic health group, two models are presented: an unadjusted model which contains only the BMI category–metabolic health group and depressive symptoms variables, and an adjusted model that contains these same variables and the identified confounding variables. Multiple imputation was used to ascribe missing exposure, confounder, and outcome data, based on the assumption that the data are missing at random, and an unadjusted and adjusted effect size is presented for the complete case and the multiple imputed analyses. The assumptions of each multiple logistic regression model were verified by using a P-P plot, the scatter plots of residuals, and a Variance Inflation Factor (VIF) to check for the linearity of the independent variable and log-odds, no strongly influential outliers, an absence of multicollinearity, and an appropriate outcome type. 

An interaction analysis was also performed to determine whether the odds of depressive symptoms were different in each combination of a BMI category and metabolic health group. 

Multiple logistic regression was used to compare the odds of moderate to severe depressive symptoms in each BMI category for metabolically healthy and metabolically unhealthy groups using ‘BMI category × metabolic health group’ as an interaction term in the model. Participants categorised as normal weight and metabolically healthy were considered the reference group because they have the lowest odds of experiencing depressive symptoms. An OR and 95% CI were estimated for each BMI category and metabolic health group and a *p*-value < 0.05 was considered statistically significant. The within-strata OR was calculated by dividing the OR from each BMI category and metabolic health group within that metabolic health strata by the OR from the normal weight group for both metabolically healthy and metabolically unhealthy respondents. The relative excess risk due to interaction (RERI), along with the 95% CI and *p*-value, on an additive scale were calculated for each potential interaction effect using STATA 18 (Stata Corp. 2023. Stata Statistical Software. Release 18. College Station, TX, USA: Stata Corp LLC). 

To determine whether the odds of moderate to severe depressive symptoms differed according to gender [i.e., effect modification], multiple logistic regression was used to compare the odds of depressive symptoms in each BMI category–metabolic health group for males and females using ‘gender × BMI category–metabolic health group’ as an interaction term in the model. Male participants categorised as MHNW were considered the reference group as they have the lowest odds of experiencing depressive symptoms. An OR and 95% CI were estimated for each BMI category–metabolic health group and a *p*-value < 0.05 was considered statistically significant. The within-strata OR was calculated by dividing the OR from each BMI category–metabolic health group within the gender strata by the OR from the MHNW group for both males and females. 

## 3. Results

**Figure 1:**
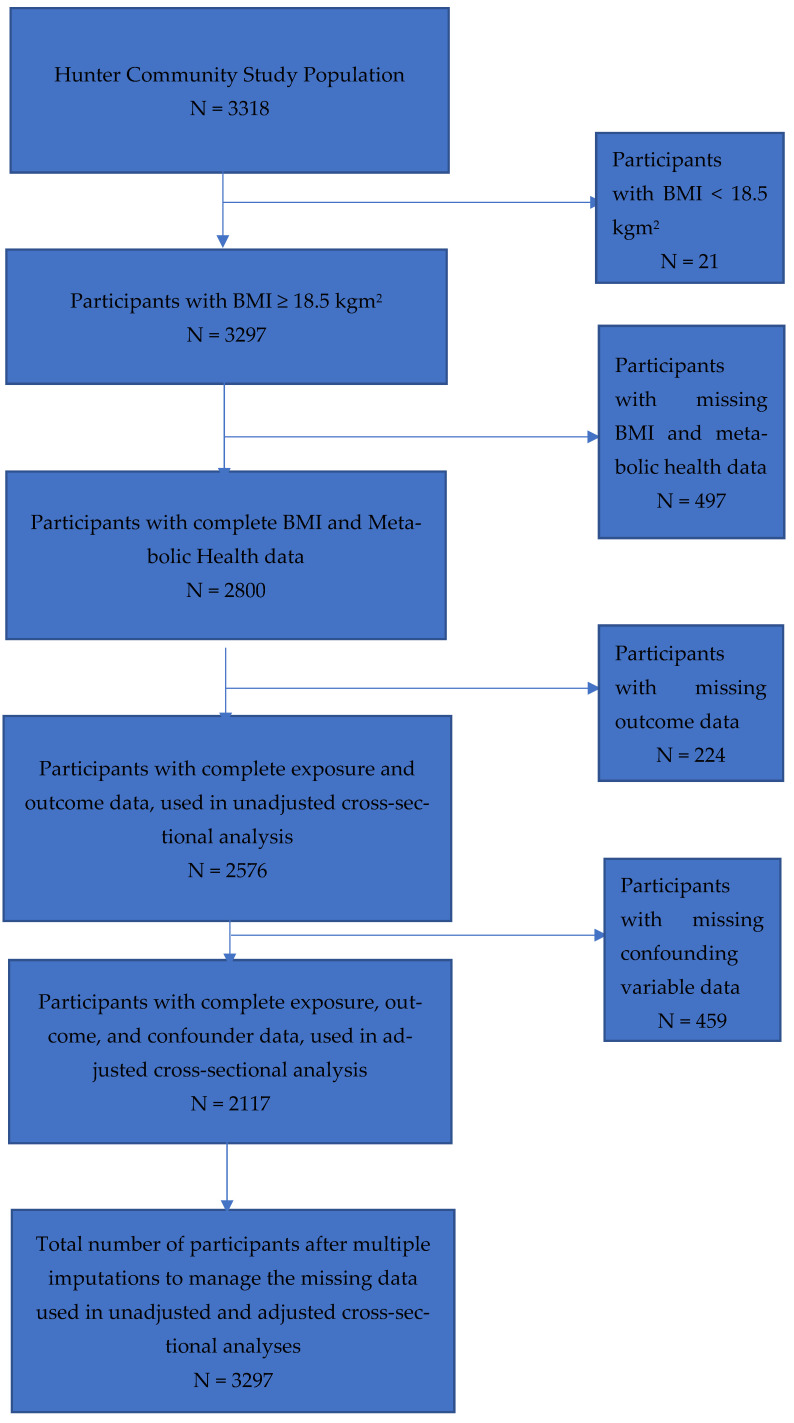
Flow diagram for the selection of participants from the Hunter Community Study with complete data, for six obesity phenotypes and confounders after the exclusion of participants with BMI < 18.5 kgm^2^, missing BMI/metabolic health data, missing outcome and confounder data, and multiple imputations to manage the missing data.

There were 3318 participants enrolled in the HCS cohort. There were 21 participants excluded because their BMI was less than 18.5 kgm^2^ (underweight). Of the 3297 remaining participants with a BMI of 18.5 kgm^2^ or more, 497 had missing BMI and metabolic health data, leaving 2800 participants with complete BMI and metabolic health data. There were 224 participants with missing outcome data, leaving 2576 participants with complete outcome and exposure data, who were included in the unadjusted complete-case cross-sectional analysis. There were 2117 participants with complete exposure, outcome, and confounding variable data that were included in the adjusted complete-case cross-sectional analysis, as there were 459 participants with missing confounding variable data [see Figure 1]. Missing data were imputed, resulting in 3297 participants included in the final imputed analysis.

### 3.1. Baseline Characteristics of the Study Population

The baseline characteristics of the study population, categorised according to BMI category–metabolic health group, are presented in Table 1. Approximately 21.9% and 11.2% of the total population were classified as MHOW and MHO, respectively. Participants categorised as having obesity constituted about 34.0% of the sample, with 32.8% of this sub-group classified as MHO. Approximately 14.0% of the sample had moderate to severe depressive symptoms, measured using the CES-D scale. 

The average age of the participants was 66.2 years (SD 7.5), with the groups categorised as metabolically healthy having a lower mean age than their counterparts that were categorised as unhealthy. Slightly more than half of the sample was female (i.e., 51.5%) with about 44.0% classified as metabolically unhealthy and either overweight or obese. 

### 3.2. BMI Category–Metabolic Health Groups and Their Odds of Experiencing Depressive Symptoms

For the primary analysis, only the findings from the imputed dataset are presented as these provide a more valid estimate of the results. For comparison, the findings from the complete (unimputed) case analysis are presented in Supplementary Table S1.

### 3.3. Unadjusted Models

In the multiple imputation analysis, compared with the MHNW group, the unadjusted OR for moderate to severe depressive symptoms in the MHOW group was 0.83 (95% CI: 0.53–1.28, 0.39), and in the MHO group it was 1.25 (95% CI: 0.81–1.93, 0.32). The OR for moderate to severe depressive symptoms was increased in the MUNW group (OR = 1.34, 95% CI: 0.82–2.17, 0.24) and the MUO group (OR = 1.84, 95% CI: 1.27–2.67, 0.001) but not in the MUOW group (OR = 1.02, 95% CI: 0.68–1.52, 0.93) (see Table 2).

### 3.4. Adjusted Models

In a multiple imputation analysis, compared with the MHNW group, the adjusted OR for moderate to severe depressive symptoms in the MHOW group was 0.80 (95% CI: 0.51–1.26, 0.34), and in the MHO group it was 1.03 (95% CI: 0.65–1.64, 0.89). The adjusted OR for moderate to severe depressive symptoms was increased in the MUNW group (OR = 1.25, 95% CI: 0.76–2.06, 0.38) and the MUO group (OR = 1.48, 95% CI: 1.00–2.19, 0.05) but not in the MUOW group (OR = 0.96, 95% CI: 0.63–1.45, 0.85) (see Table 2). 

### 3.5. Results of the Odds of Experiencing Moderate to Severe Depressive Symptoms According to the Interaction between BMI and Metabolic Health Category

Given that the odds of moderate to severe depressive symptoms appear to be only increased in those who were metabolically unhealthy, we also formally tested the odds of experiencing moderate to severe depressive symptoms due to interaction between a person’s BMI category and their metabolic health group (see Table 3). ORs, with 95% CIs and *p*-values, are presented for the only metabolically unhealthy group (1.27; 0.76–2.13, 0.36), for the only overweight group (0.77; 0.50–1.19, 0.24) and for the metabolically unhealthy and overweight group (0.94; 0.62–1.44, 0.80), where the metabolically healthy group with normal weights is the reference category. ORs, with 95% CIs and *p*-values, are presented for the association between metabolic health and moderate to severe depressive symptoms in the different strata of BMI categories—normal weight (1.27; 0.76–2.13, 0.36) and overweight (1.23; 0.84–1.80, 0.29). ORs, with 95% CIs and *p*-values, are also presented for the association between BMI overweight categories and moderate to severe depressive symptoms in the strata of metabolic health (metabolically healthy group: 0.77; 0.50–1.19, 0.24. Metabolically unhealthy group: 0.74; 0.47–1.19, 0.32).

For the overweight and metabolically unhealthy group, their RERI, 95% CI, and *p*-value were −0.11, −0.92–0.70, and 0.79. These suggest that the estimated joint effect, on an additive scale, of the overweight and metabolically unhealthy group was less than the sum of the estimated effects of the overweight alone and metabolically unhealthy alone groups, i.e., there was some indication of their negative interaction on an additive scale; However, this was not statistically significant.

ORs, with 95% CIs and *p*-values, are presented for the only metabolically unhealthy group (1.27; 0.76–2.13, 0.36), for the obesity only group (0.98; 0.61–1.57, 0.93) and for the metabolically unhealthy group with obesity (1.45; 0.96–2.20, 0.08), where the metabolically healthy group with normal weight was the reference category. ORs, with CIs and *p*-values, are presented for the association between metabolic health and moderate to severe depressive symptoms in the strata of BMI categories–normal weight (1.27; 0.76–2.13, 0.36) and obesity (1.48; 1.00–2.20, 0.05). ORs, with CIs and *p*-values, are also presented for the association between BMI obesity category and moderate to severe depressive symptoms in the strata of metabolic health (metabolically healthy group: 0.98; 0.61–1.57, 0.93. Metabolically unhealthy group: 1.14; 0.71–1.82, 0.59).

For the obesity and metabolically unhealthy group, their RERI, CI, and *p*-value were 0.25, −0.47–0.96, and 0.50. This suggests that the estimated joint effect, on an additive scale, of obesity and being metabolically unhealthy together may be greater than the sum of the estimated effects of obesity alone and being metabolically unhealthy alone, i.e., there is some indication of their positive interaction on an additive scale; however, this was not statistically significant.

### 3.6. Association between BMI Category–Metabolic Health Group and Moderate to Severe Depressive Symptoms According to Gender

In a multiple-imputation-adjusted analysis, and using males categorised with MHNW as the reference group, the odds of experiencing moderate to severe depressive symptoms in males were increased in all BMI category–metabolic health groups. However, these ORs were statistically significant only in males in the MUO group (OR = 2.54, 95% CI: 1.15–5.64, 0.02) but not in the MHOW (OR = 1.14, 95% CI: 0.50–2.61, 0.76), MHO (OR = 2.01, 95% CI: 0.84–4.82, 0.12), MUNW (OR = 1.31, 95% CI: 0.43–4.00, 0.63), or MUOW groups (OR = 1.46, 95% CI: 0.65–3.27, 0.36) (see Table 4). Compared with the male MHNW reference group, the odds of experiencing moderate to severe depressive symptoms in females were increased in all BMI category–metabolic health groups. However, these ORs were statistically significant only in the MHNW (OR = 2.45, 95% CI: 1.07–5.61, 0.03), MUNW (OR = 2.88, 95% CI: 1.21–6.84, 0.02), and MUO groups (OR = 2.92, 95% CI: 1.31–6.49, 0.01) but not the MHOW (OR = 1.69, 95% CI: 0.74–3.89, 0.22), MHO (OR = 1.76, 95% CI: 0.73–4.24, 0.21), or MUOW groups (OR = 1.99, 95% CI: 0.87–4.51, 0.10); the tests for effect modification were chi2 = 21.99, *p*-value = 0.025 (see Table 4).

### 3.7. Within-Strata Effect of Gender on the Odds of Experiencing Moderate to Severe Depressive Symptoms

The odds of moderate to experiencing severe depressive symptoms for each gender, with reference group being the MHNW group of the respective gender, are also presented in Table 4. Compared with the male MHNW group, the odds of experiencing moderate to severe depressive symptoms for males only were similarly increased in all other BMI category–metabolic health groups. Compared with the female MHNW group, the odds of experiencing moderate to severe depressive symptoms for females only were increased in the MUNW (OR = 1.17, 95% CI: 0.64–2.14, 0.60) and MUO groups (OR = 1.19, 95% CI: 0.72–1.95, 0.50) but not in the MHOW (OR = 0.68, 95% CI: 0.39–1.18, 0.17), MHO (OR = 0.71, 95% CI: 0.38–1.32, 0.28), or MUOW groups (OR = 0.81, 95% CI: 0.47–1.38, 0.44). The effect estimates were all not statistically significant within female groups.

## 4. Discussion

This cross-sectional study evaluated the association between overweight/obesity, metabolic health, and moderate to severe depressive symptoms in older adults within the HCS cohort. The proportion of adults with MHOW and MHO was 21.9% and 11.2%, respectively, and was consistent with the findings from other studies of adult populations reported in the literature [39]. The prevalence of depression in our study population was about 14% and is comparable to the findings from other studies of a similar population group [20,22]. In those categorised as MHOW and MHO, there were no differences in their odds of experiencing moderate to severe depressive symptoms compared to those categorised as MHNW. The adjusted odds of experiencing moderate to severe depressive symptoms were 25% higher in adults categorised as MUNW and 48% higher in those classified as MUO compared with the MHNW reference group; however, these effect estimates were not statistically significant. 

Similar to our study, a systematic review, which included ten cross-sectional studies and two cohort studies, examined the BMI category and metabolic health status of participants and their odds of experiencing moderate to severe depressive symptoms, assessed by depressive symptom scales including the CES-D scale, and found no association between their MHO phenotype and moderate to severe depressive symptoms [23]. Several other studies also found that MHO was not associated with an increased risk of moderate to severe depressive symptoms assessed using the CES-D scale [20,21,22,40,41,42]; however, a meta-analysis of eight cross-sectional studies found an increased odds ratio of moderate to severe depressive symptoms, assessed by standardised rating scales including the CES-D scale, in MHO (OR = 1.32, 95% CI: 1.13–1.54) [24]. The only published systematic review (*N* = 65,163) that has examined the association between BMI category–metabolic health groups and depressive symptoms found an increased risk of moderate to severe depressive symptoms, assessed by depressive symptom scales including the CES-D scale, in adults categorised as MUNW or MUO [23]. Similar results have also been reported more recently, including in three cohort and two cross-sectional studies [20,21,22,43,44]. Our study also found an increased odds ratio of experiencing moderate to severe depressive symptoms when MUO or MUNW, but this was not statistically significant, probably due to our cohort size. Our study found some indication of an interaction, on an additive scale, between overweight and obesity and metabolic health; however, this was not statistically significant. The associations between moderate to severe depressive symptoms and overweight/obesity and metabolic health appear to be primarily due to an unhealthy metabolic status.

Based on previous conflicting research findings, this study had an a priori hypothesis that the odds of experiencing moderate to severe depressive symptoms would differ between males and females across different BMI category–metabolic health groups. Several studies have examined the odds of experiencing depressive symptoms, assessed using the CES-D scale, across BMI category–metabolic health groups [20,21,22,40,41,42,43,44], and some have examined gender differences [40,43,44]. However, these studies used subgroup analyses only, rather than the preferred approach of exploring the effect of gender through an interaction term in a regression model. This study found that, across all six BMI category–metabolic health groups, there is an increased odds ratio of experiencing moderate to severe depressive symptoms in females compared to the MHNW male reference group. Hence, the odds of experiencing depressive symptoms appear to be dependent on gender. This finding is consistent with international psychiatric epidemiological studies, which have consistently reported an increased odds/risk of clinically significant depressive symptoms in females compared to males [44,45,46]. The gender differences in depressive symptoms may be due to a number of factors including biological, social, and developmental factors [47]. However, this difference might also be due to reporting bias, where some men report few symptoms because they perceive it to be socially undesirable [48].

In a subgroup analysis from a large cohort study of older adults (*N* = 3,586,492), the odds of depression defined as a moderate or severe depressive episode, assessed using ICD-10 codes F32.0–F34.9, was increased in females categorised as MUNW, MHO, or MUO and males categorised as MUO, as reported by Seo et al. [44]. A similar association between four BMI category–metabolic health groups and moderate to severe depressive symptoms, measured using the Patient Health Questionnaire-9 (PHQ9), was observed in a large cross-sectional study of older adults (*N* = 18,025) [43]. Our study demonstrated increased the odds of experiencing moderate to severe depressive symptoms in females with MHNW, MUNW, or MUO and males with MUO; these findings were statistically significant when compared to the male MHNW reference group. The odds of these groups of females experiencing moderate to severe depressive symptoms were higher than those in corresponding groups of males. However, there was an indication of the increasing odds of experiencing moderate to severe depressive symptoms across BMI categories in males regardless of their metabolic health status. This may be due to other factors which increase the risk of them developing moderate to severe depressive symptoms, such as poor diet, inactivity, and co-occurrence with alcohol use [49]. However, our study population is older compared to the study population in these two studies. The relationship between moderate to severe depression and overweight/obesity in this study appears to be driven by metabolic health status, signalling the need for attention to metabolic health in addition to weight and mental health management of people diagnosed with depression who also have overweight/obesity. There is also need for a longitudinal study to explore these relationships. The gender difference in the relationship between depression, overweight/obesity, and metabolic health points to the importance of further research into gender-related factors which may contribute to the different depression outcomes in males and females with overweight or obesity.

The relationship between obesity and moderate to severe depressive symptoms may be mediated by many factors which are associated with systemic inflammation. These factors include psychosocial stress, poor diet, physical activity, obesity, smoking, altered gut permeability, atopy, dental caries, sleep, and vitamin D deficiency [49]. The association between moderate to severe depressive symptoms and the six BMI category–metabolic health groups in our study appears to be driven primarily by metabolic health rather than BMI category. There may be increased odds of experiencing moderate to severe depressive symptoms in metabolically unhealthy compared to metabolically healthy older adults. The potential mechanism of this may be related to the presence of metabolic risk factors which are associated with inflammation. Systemic inflammation has been reported to be a strong contributor to obesity-related depressive symptoms [50]. This may be mediated through immuno-inflammatory mediators, altered neuroendocrine functions, the hypothalamic–pituitary–adrenal axis, leptin, insulin, and the microbiota [51]. The inclusion of inflammation in the definition of being metabolically unhealthy may explain the association between poor metabolic health and clinically significant depressive symptoms, and future research should examine this further. 

## 5. Strengths and Limitations

To our knowledge, this is the first study to examine the association between BMI–metabolic health phenotypes and depressive symptoms in adults in Australia. The important strengths of this study are the use of a well-validated, objective measure to identify participants with and without depressive symptoms. This study categorised BMI and metabolic health into six categories, which reduces the risk of misclassification bias, which can occur due to the overlap of different overweight/obesity groups in studies where participants are grouped into those with obesity and those without obesity. The sample was derived from a well-designed cohort study that is mostly representative of the community-dwelling older adults in Australia. 

The relatively small sample size within each of the overweight/obesity–metabolic health groups is a limitation which has produced imprecise effect estimates and may be responsible for the lack of statistical significance in some of our findings. However, the magnitude of the effect size provides an estimate of the true effect, and these findings should be pooled with findings from other cross-sectional studies to increase the precision of these effect estimates. 

The cross-sectional study design means that the temporal association between BMI–metabolic health groups and the development/risk of moderate to severe depressive symptoms could not be established. The increased odds of experiencing moderate to severe depressive symptoms in the MUNW and MUO groups in our study, though not statistically significant, implies a role of metabolic health in driving depressive symptoms in adults with obesity. However, moderate to severe depressive symptoms may lead to inactivity, poor diet, and increased alcohol use, resulting in development of metabolic dysfunction [46,52,53]. Given that obesity, metabolic health, and depression are among the most prevailing health challenges across the globe, there is need for longitudinal research that examines the relationship between BMI–metabolic health phenotypes and the risk of clinically significant depressive symptoms and considers the changes in BMI, inflammatory, and metabolic health status over time.

## 6. Conclusions

This study demonstrated an increased odds ratio of experiencing moderate to severe depressive symptoms in metabolically unhealthy older adults regardless of their BMI category. However, these effects were not statistically significant. Females had an increased odds ratio of experiencing moderate to severe depressive symptoms across all BMI–metabolic health groups, compared with a metabolically healthy normal weight male reference group. There was a trend of increased odds of experiencing moderate to severe depressive symptoms in males across all BMI–metabolic health groups, with the greatest odds in males with MUO. Future research should examine the potential link between metabolic health and depressive symptoms. 

## Figures and Tables

**Table 1:** Characteristics of study population according to their BMI categories and metabolic health groups.

	Normal Weight		Overweight		Obesity		Total
	Metabolically Healthy	Metabolically Unhealthy	Metabolically Healthy	Metabolically Unhealthy	Metabolically Healthy	Metabolically Unhealthy	
Percentage and number of participants	12.3% (316)	7.8% (201)	21.9% (564)	23.9% (616)	11.2% (288)	22.9% (591)	100% (2576)
Females (%)	62.7	65.5	45.1	44.3	55.8	51.8	51.5
Age (mean, SD) (median, IQR)	65.3 (7.9) 63.1 (11.3)	68.9 (7.7) 68.6 (11.4)	64.8 (7.0) 63.1 (10.2)	67.9 (7.7) 67.0 (12.8)	63.8 (6.6) 62.0 (9.1)	66.5 (7.0) 65.6 (10.9)	66.2 (7.5) 64.8 (11.7)
Percentage and number of participants with moderate to severe depressive symptoms at baseline in each group	12.3% (*N* = 39)	14.4% (*N* = 29)	9.9% (*N* = 56)	11.7% (*N* = 72)	14.6% (*N* = 42)	19.6% (*N* = 116)	13.7% (*N* = 354)
BMI (mean, SD)	23.0 1.5	23.1 1.5	27.3 1.4	27.6 1.4	33.4 3.6	34.4 4.2	28.8 4.9
HDL-Chol (mean, SD)	1.6 0.4	1.5 0.4	1.4 0.4	1.3 0.3	1.4 0.4	1.2 0.3	1.4 0.4
Trig (mean, SD)	1.0 0.4	1.2 0.6	1.2 0.6	1.5 0.9	1.4 0.9	1.8 1.4	1.4 1.0
Fasting glucose (mean, SD)	4.7 0.6	4.9 1.0	4.8 0.6	5.3 1.4	4.9 0.6	5.7 1.6	5.1 1.2
SBP (mean, SD)	129.7 18.6	137.5 20.3	134.3 18.1	139.5 19.6	133.7 16.7	136.8 17.4	135.7 18.7
DBP (mean, SD)	76.4 9.6	76.7 9.5	79.8 9.5	79.1 10.1	79.9 9.3	78.3 10.5	78.6 9.9
Number of alcoholic drinks/day (mean, SD)	1.8 2.1	1.3 2.1	2.1 2.5	2.2 3.6	2.1 2.7	2.0 2.8	2.0 2.8
Mean daily step count (mean, SD)	8377.9 2975.1	7258.7 3092.5	7285.1 3100.1	6802.0 3238.2	6274.2 2835.8	5580.9 2951.4	6815.6 3174.2
Highest level of education: primary or secondary (percentage, numbers)	45.0% (157)	47.1% (105)	39.8% (236)	47.7% (317)	46.5% (146)	53.3% (335)	46.7% (1296)
Highest level of education: university or tertiary-level (percentage, numbers)	55.0% (192)	52.9% (118)	60.2% (357)	52.3% (348)	53.5% (168)	46.7% (294)	53.3% (1477)
Smoking status: never (percentage, numbers)	206 (59.7%)	131 (58.7%)	321 (54.0%)	350 (53.0%)	164 (52.4%)	338 (53.5%)	1510 (54.5%)
Smoking status: ever (percentage, numbers)	107 (31.0%)	73 (32.7%)	223 (37.5%)	279 (42.2%)	123 (39.3%)	250 (39.6%)	1055 (38.1%)
Smoking status: current (percentage, numbers)	32 (9.3%)	19 (8.5%)	51 (8.6%)	32 (4.8%)	26 (8.3%)	44 (7.0%)	204 (7.4%)

Normal weight: BMI = 18.5–24.9 kgm^2^; overweight: BMI = 25–29.9 kgm^2^; obesity: BMI ≥ 30 kgm^2^. Metabolically healthy: presence of no metabolic risk factors; metabolically unhealthy: presence of one or more metabolic risk factors. Categorical variables are presented as percentages (%). SD = Standard deviation. BMI = Body Mass Index (kg/m^2^). SBP = Systolic Blood Pressure (mmHg). DBP = Diastolic Blood Pressure (mmHg). Trig = Triglycerides (mm/L). HDL-Chol = high-density lipoprotein cholesterol (mm/L). Fasting glucose (mmol/L). Education was classified, according to the highest level of education attained, as primary and secondary schooling completed or trade qualification, university, and other tertiary-level study completed. Physical activity was measured by recorded step counts using a pedometer worn by participants for seven consecutive days during waking hours and was reported as the mean number of steps per day. Smoking frequency was measured by the number of cigarettes per day and reported as never-, ever-, or current smoker. Alcohol consumption was measured by the number of standard drinks per day, defined according to Australian National Health and Medical Research Council (NHMRC) guidelines and reported as the mean number of standard alcohol drinks per day.

**Table 2:** Cross-sectional imputed data analysis of the effects of BMI–metabolic health group on moderate/severe depressive symptoms.

		Normal Weight		Overweight		Obesity	
		Metabolically Healthy	Metabolically Unhealthy	Metabolically Healthy	Metabolically Unhealthy	Metabolically Healthy	Metabolically Unhealthy
Percentage and number of participants in the complete case analysis		12.3% (*N* = 316)	7.8% (*N* = 201)	21.9% (*N* = 564)	23.9% (*N* = 616)	11.2% (*N* = 288)	22.9% (*N* = 591)
Percentage and number of participants with moderate/severe depressive symptoms at baseline in each group		12.3% (*N* = 39)	14.4% (*N* = 29)	9.9% (*N* = 56)	11.7% (*N* = 72)	14.6% (*N* = 42)	19.6% (*N* = 116)
Unadjusted multiple imputation model	OR 95% CI *p*-value	1	1.34 0.82–2.17 0.24	0.83 0.53–1.28 0.39	1.02 0.68–1.52 0.93	1.25 0.81–1.93 0.32	1.84 1.27–2.67 0.001
Adjusted multiple imputation model *	OR 95% CI *p*-value	1	1.25 0.76–2.06 0.38	0.80 0.51–1.26 0.34	0.96 0.63–1.45 0.85	1.03 0.65–1.64 0.89	1.48 1.00–2.19 0.05

Normal weight: BMI = 18.5–24.4 kgm^2^; overweight: BMI = 25–29.9 kgm^2^; obesity: BMI ≥ 30 kgm^2^. Metabolically healthy: presence of no metabolic risk factor; metabolically unhealthy: presence of one or more metabolic risk factors. OR = odds ratio, CI = Confidence Interval, *p*-value = level of significance (<0.05). * Adjusted for gender, age, highest education level, mean daily standard alcohol drinks per day, mean weekly steps, smoking status, diet quality.

**Table 3:** Odds of experiencing moderate/severe depressive symptoms according to the interaction between each BMI category and metabolic health.

BMI Category	Metabolically Healthy				Metabolically Unhealthy				
	OR	95% CI	*p*-Value	OR, 95% CI, *p*-Value within the Strata of Metabolically Healthy	OR	95% CI	*p*-Value	OR, 95% CI, *p*-Value within the Strata of Metabolically Unhealthy	OR, 95% CI, *p*-Value within the Strata of BMI Categories
Normal Weight	1	-	-	1	1.27	0.76–2.13	0.36	1	1.27 0.76–2.13 0.36
Overweight	0.77	0.50–1.19	0.24	0.77 0.50–1.19 0.24	0.94	0.62–1.44	0.80	0.74 0.47–1.19 0.32	1.23 0.84–1.80 0.29
Obesity	0.98	0.61–1.57	0.93	0.98 0.61–1.57 0.93	1.45	0.96–2.20	0.08	1.14 0.71–1.82 0.59	1.48 1.00–2.20 0.05

BMI = Body Mass Index. Normal weight: BMI = 18.5–24.4 kgm^2^; overweight: BMI = 25–29.9 kgm^2^; obesity: BMI ≥ 30 kgm^2^. Metabolically healthy: presence of no metabolic risk factor; Metabolically unhealthy: presence of one or more metabolic risk factors. OR = odds ratio, CI = Confidence Interval, *p*-value = level of significance (<0.05). Reference group for OR across strata: metabolically healthy normal weight, OR = 1. Reference group for OR within metabolically healthy strata: metabolically healthy normal weight, OR = 1. Reference group for OR within metabolically unhealthy strata: metabolically unhealthy normal weight, OR = 1. OR within strata of BMI category: calculated by dividing OR for metabolically unhealthy group by OR for metabolically healthy group in the same BMI strata. Metabolic health/Overweight: RERI = −0.11, CI: −0.92–0.71, *p*-value = 0.79. Metabolic health/Obesity: RERI = 0.25, CI: −0.47–0.96, *p*-value = 0.50.

**Table 4:** Odds of experiencing moderate/severe depressive symptoms according to BMI and metabolic health category—results according to male and female gender (imputed data analysis).

	Male			Female		
BMI–Metabolic Health Category	N with/without Depressive Symptoms	OR (95% CI)	ORs within Male Strata	N with/without Depressive Symptoms	OR (95% CI)	ORs within Female Strata
MHNW *N* = 316	8/107	1	1	31/170	2.45 (1.07–5.61)	1
MHOW *N* = 564	27/281	1.14 (0.50–2.61)	1.14 (0.50–2.61)	29/227	1.69 (0.74–3.89)	0.68 (0.39–1.18)
MHO *N* = 288	22/110	2.01 (0.84–4.82)	2.01 (0.84–4.82)	20/136	1.76 (0.73–4.24)	0.71 (0.38–1.32)
MUNW *N* = 201	6/62	1.31 (0.43–4.00)	1.31 (0.43–4.00)	23/110	2.88 (1.21–6.84)	1.17 (0.64–2.14)
MUOW *N* = 616	37/314	1.46 (0.65–3.27)	1.46 (0.65–3.27)	35/230	1.99 (0.87–4.51)	0.81 (0.47–1.38)
MUO *N* = 591	54/232	2.54 (1.15–5.64)	2.54 (1.15–5.64)	62/243	2.92 (1.31–6.49)	1.19 (0.72–1.95)

Normal weight: BMI = 18.5–24.4 kgm^2^; overweight: BMI = 25–29.9 kgm^2^; obesity: BMI ≥ 30 kgm^2^. Metabolically healthy: presence of no metabolic risk factor; metabolically unhealthy: presence of one or more metabolic risk factors. OR = odds ratio, CI = Confidence Interval, *p*-value = level of significance (<0.05). Significant *p*-values: MUO (male) OR = 2.54, CI: 1.15–5.64, *p*-value = 0.02. MUO (female) OR = 2.92, CI: 1.31–6.49, *p*-value = 0.01. MHNW (female) OR = 2.45, CI: 1.07–5.61, *p*-value = 0.03. MUNW (female) OR = 2.88, CI: 1.21–6.84, *p*-value = 0.02. Reference group for OR females compared with males = males classified as MHNW. Reference group for OR within male strata: males classified as MHNW. Reference group for OR within female strata = females classified as MHNW. MHNW = metabolically healthy normal weight, MHOW = metabolically healthy overweight, MHO = metabolically healthy obesity, MUNW = metabolically unhealthy normal weight, MUOW = metabolically unhealthy overweight, MUO = metabolically unhealthy obesity.

## Data Availability

The data presented in this study are available on request from the corresponding author due to privacy and ethical reasons.

## Supplementary Materials

The following supporting information can be downloaded at https://www.mdpi.com/article/10.3390/nu16070928/s1, Table S1: Cross-sectional complete case analysis for the effects of BMI-metabolic health group and moderate/severe depressive symptoms.

## Abbreviations

ARFS	Australian Recommended Food Score.
BMI	Body Mass Index.
CES-D	Centre for Epidemiological Studies Depression Scale.
CI	Confidence Interval.
DAG	directed acyclic graph.
DSM-IV	Diagnostic and Statistical Manual of Mental Disorders, 4th Edition.
IDF	International Diabetes Federation.
MHNW	metabolically healthy normal weight.
MHOW	metabolically healthy overweight.
MHO	metabolically healthy obesity.
MUNW	metabolically unhealthy normal weight.
MUOW	metabolically unhealthy overweight.
MUO	metabolically unhealthy obesity.
NCEP ATP-III	National Cholesterol Education Program Adult Treatment Panel-III report.
OR	odds ratio.
RERI	relative excess risk due to interaction.

## Appendix A

Directed acyclic graph (DAG) of potential confounding variables for the effect of Metabolic health–BMI status on moderate to severe depressive symptoms.

Exposure: metabolically healthy obesity (MHO).

Outcome: depressive symptoms.

Minimal sufficient adjustment set included age, gender, physical activity, education, diet quality, alcohol intake, and smoking.
