# Supplementary material for: Metabolic Health, Overweight or Obesity, and Depressive Symptoms among Older Australian Adults
Source: Nutrients. 2024 Mar 23;16(7):928. doi: 10.3390/nu16070928 (PMC11013641; doi:10.3390/nu16070928)
Supplement: Supplementary file 1 [file nutrients-16-00928-s001.zip › nutrients-2895037-supplementary.pdf]

Supplementary Table S1: Cross-sectional complete case analysis for the effects of BMI - metabolic health group and moderate/severe depressive symptoms.

		Normal Weight		Overweight		Obesity	
		**Metabolically Healthy**	**Metabolically Unhealthy**	**Metabolically Healthy**	**Metabolically Unhealthy**	**Metabolically Healthy**	**Metabolically Unhealthy**
Percentage and number of participants Complete case analysis		12.3% (N = 316)	7.8% (N = 201)	21.9% (N = 564)	23.9% (N = 616)	11.2% (N = 288)	22.9% (N = 591)
Percentage and number with moderate/severe depressive symptoms at baseline		12.3% (N = 39)	14.4% (N = 29)	9.9% (N = 56)	11.7% (N = 72)	14.6% (N = 42)	19.6% (N = 116)
Unadjusted Complete Case analysis	OR<br>95% CI<br>*p*-value	1	1.20<br>0.71–2.01<br>0.49	0.78<br>0.51–1.21<br>0.27	0.94<br>0.62–1.42<br>0.77	1.21<br>0.76–1.94<br>0.42	1.73<br>1.17–2.57<br>0.006
Adjusted Complete Case analysis*	OR<br>95% CI<br>*p*-value	1	1.29<br>0.73–2.30<br>0.38	0.72<br>0.44–1.20<br>0.21	0.92<br>0.57–1.49<br>0.72	0.74<br>0.41–1.31<br>0.29	1.25<br>0.78–2.01<br>0.35

Normal weight: BMI = 18.5–24.4 kgm^2; Overweight: BMI = 25–29.9kgm^2; Obesity: BMI ≥ 30 kgm^2

Metabolically Healthy: presence of no metabolic risk factor; Metabolically Unhealthy: presence of one or more metabolic risk factors.

OR = Odds Ratio, CI = Confidence Interval, *p*-value = level of significance (<0.05).

* Adjusted for age, gender, highest education level, mean daily standard alcohol drinks per day, mean weekly steps, smoking status, diet quality
